# The Social and Spatial Ecology of Dengue Presence and Burden during an Outbreak in Guayaquil, Ecuador, 2012

**DOI:** 10.3390/ijerph15040827

**Published:** 2018-04-23

**Authors:** Catherine A. Lippi, Anna M. Stewart-Ibarra, Ángel G. Muñoz, Mercy J. Borbor-Cordova, Raúl Mejía, Keytia Rivero, Katty Castillo, Washington B. Cárdenas, Sadie J. Ryan

**Affiliations:** 1 Quantitative Disease Ecology and Conservation Lab, Department of Geography, University of Florida, Gainesville, FL 32611 USA; clippi@ufl.edu; 2 Emerging Pathogens Institute, University of Florida, Gainesville, FL 32608, USA; 3 Center for Global Health and Translational Science and Department of Medicine, State University of New York Upstate Medical University, Syracuse, NY 13210, USA; stewarta@upstate.edu; 4 Atmospheric and Oceanic Sciences (AOS), Princeton University, Princeton, NJ 08540, USA; agmunoz@iri.columbia.edu; 5 International Research Institute for Climate and Society (IRI), Earth Institute, Columbia University, New York, NY 10964, USA; 6 Escuela Superior Politécnica del Litoral (ESPOL), Guayaquil 09015863, Ecuador; meborbor@espol.edu.ec; 7 National Institute of Meteorology and Hydrology (INAMHI), Quito 170135, Ecuador; rmejia@inamhi.gob.ec (R.M.); keytiar@gmail.com (K.R.); 8 Institute of Biometrics and Epidemiology, Auf’m Hennekamp 65, 40225 Düsseldorf, Germany; katty.castillo@ddz.uni-duesseldorf.de; 9 Laboratorio de Biomedicina, FCV, Escuela Superior Politécnica del Litoral (ESPOL), Guayaquil 09015863, Ecuador; wbcarden@espol.edu.ec

**Keywords:** dengue fever, geography, ecology, risk factors, climate, spatial analysis, temporal, Ecuador

## Abstract

Dengue fever, a mosquito-borne arbovirus, is a major public health concern in Ecuador. In this study, we aimed to describe the spatial distribution of dengue risk and identify local social-ecological factors associated with an outbreak of dengue fever in the city of Guayaquil, Ecuador. We examined georeferenced dengue cases (*n* = 4248) and block-level census data variables to identify social-ecological risk factors associated with the presence/absence and burden of dengue in Guayaquil in 2012. Local Indicators of Spatial Association (LISA), specifically Anselin’s Local Moran’s I, and Moran’s I tests were used to locate hotspots of dengue transmission, and multimodel selection was used to identify covariates associated with dengue presence and burden at the census block level. We identified significant dengue transmission hotspots near the North Central and Southern portions of Guayaquil. Significant risk factors for presence of dengue included poor housing conditions, access to paved roads, and receipt of remittances. Counterintuitive positive correlations with dengue presence were observed with several municipal services such as garbage collection and access to piped water. Risk factors for increased burden of dengue included poor housing conditions, garbage collection, receipt of remittances, and sharing a property with more than one household. Social factors such as education and household demographics were negatively correlated with increased dengue burden. These findings elucidate underlying differences with dengue presence versus burden, and suggest that vulnerability and risk maps could be developed to inform dengue prevention and control; this is information that is also relevant for emerging epidemics of chikungunya and Zika viruses.

## 1. Introduction

The public health sector in Latin America is facing the alarming situation of concurrent epidemics of dengue fever, chikungunya, and Zika, febrile viral diseases transmitted by *Aedes aegypti* and *Ae. albopictus* mosquitoes [1,2,3,4]. Traditional surveillance and vector control efforts have been unable to halt these epidemics [5]. Macro-level social-ecological factors have contributed to the global invasion, co-evolution and proliferation of the dengue viruses and vectors, including the growth of urban areas, global movement, climate change and variability, insecticide resistance, and resource-limited disease control programs. Local studies are needed to understand the complex dynamics and drivers of disease transmission, which vary from region to region, thus allowing decision makers to more effectively intervene, predict, and respond to disease outbreaks [5].

Spatial epidemiological risk maps provide important information to target focal vector control efforts in high-risk areas, potentially increasing the effectiveness of public health interventions [6,7]. Typically, historical epidemiological records are digitized to understand the spatial distribution of the burden of disease and the presence/absence of disease. Layers of social-ecological predictors (e.g., land use maps, socioeconomic census data) are incorporated to test the hypothesis that one or more predictors are associated with the presence/absence or burden of disease. Decision makers can then identify geographic areas (e.g., hotspots) to focus disease control interventions, and they can identify specific risk factors to target in these interventions (e.g., community health interventions for specific vulnerable populations). Spatial risk maps can also be integrated into disease early warning systems (EWS) to indicate areas at greater risk of epidemics during certain periods of time [8,9,10,11]. The associations between social-ecological risk factors and dengue transmission vary by place and in time, highlighting the importance of local studies of dengue transmission risk [6,12,13,14,15,16,17]. 

Since 2000, DENV 1-4 have co-circulated in Ecuador, presenting the greatest burden of disease in the lowland tropical coastal region [18]. Guayaquil, Ecuador, the focus of this study, is the largest city, and the historical epicenter of dengue transmission in the country. Chikungunya emerged in Ecuador in 2014–2015, and Zika emerged at the end of 2015; to date (4 January 2018), 6351 cases of Zika have been reported [3,19]. Climate is an important driver of variability for these diseases, mainly because both the viruses and the vectors are sensitive to temperature, and the larval development of the mosquito requires standing water (e.g. containers filled with rain or tap water). For example, climate modes associated with natural climate variability and climate change, increased the likelihood of a Zika epidemic in the Americas [4]. 

**Figure 1:**
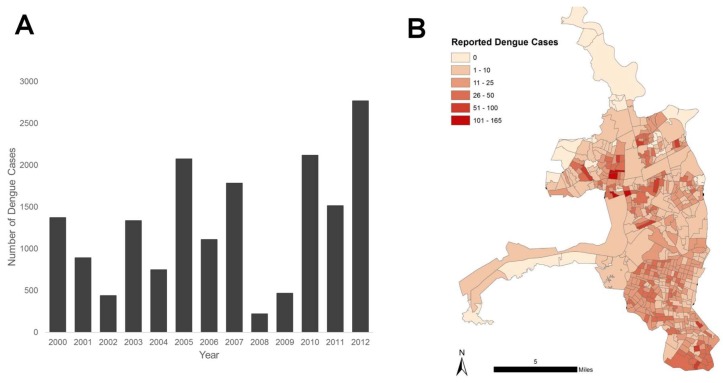
Annual number of reported cases of dengue fever in Guayaquil, Ecuador (2000–2012) (**A**). Cases of dengue fever per census zone in Guayaquil during the 2012 outbreak (**B**).

The objectives of this study were to describe the spatial dynamics and social-ecological risk factors during an epidemic of dengue fever (2012) in Guayaquil, Ecuador, when more than 4000 cases of dengue (79 dengue hemorrhagic fever-DHF) were reported, marking the biggest dengue outbreak in recent years [20]. There were 4248 clinically reported cases of dengue fever—an annual incidence of 18.07 cases per 10,000 people compared to an average annual incidence of 4.99 cases per 10,000 people from 2000 to 2011 (Figure 1) [21]. This analysis builds on prior studies in Machala, Ecuador, that demonstrated the role of social determinants in predicting dengue risk at the household and city-levels, and contributes to a broader effort to strengthen surveillance capacities in the region through collaboration with the Ministry of Health (MoH) and the National Institute of Meteorology and Hydrology (INAMHI) of Ecuador [16,17]. This study is intended to both provide the much needed local-level social-ecological context, and to demonstrate the differences arising in inference from presence and burden of cases in these analyses. 

## 2. Materials and Methods

**Figure 2:**
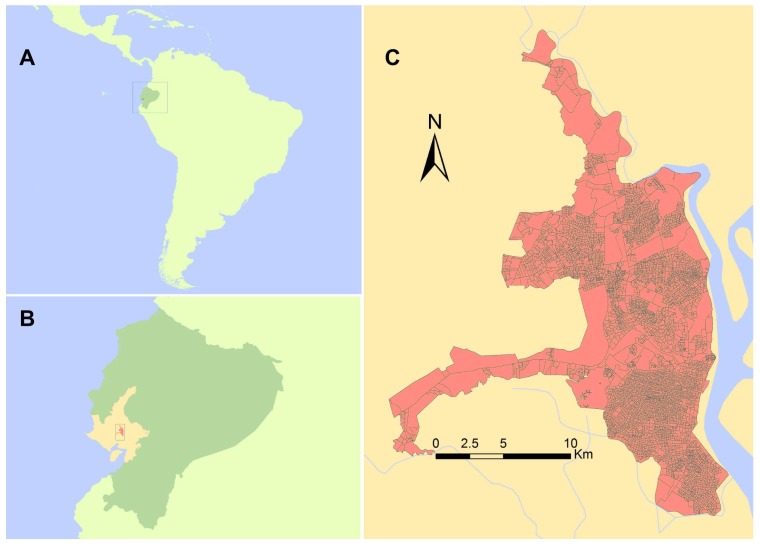
The study site, Guayaquil, is located within Guayas province in coastal Ecuador. (**Panel A**) shows the location of Ecuador (medium green) in S. America (bright green); (**Panel B**) shows the location of Guayaquil (dark orange) within Guayas Province (warm yellow), in Ecuador; (**Panel C**) shows the city of Guayaquil, detailing the census block structural layout, and showing where major and minor waterways exist surrounding the city limits.

**Figure 3:**
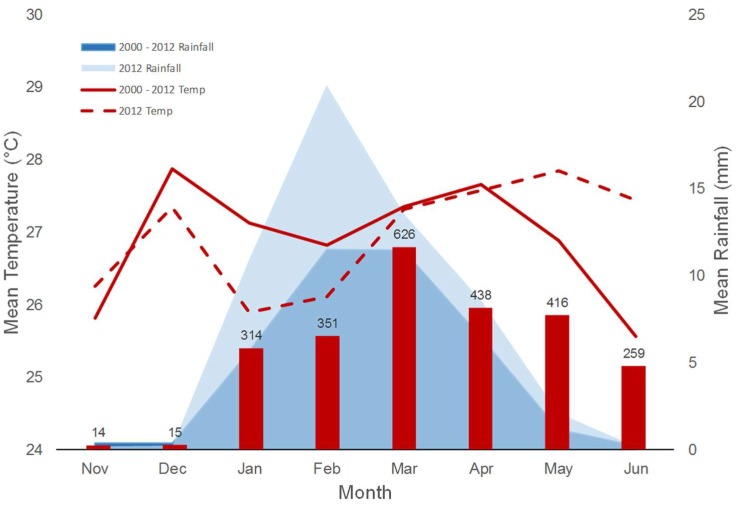
Climate seasonality and dengue cases in Guayaquil, Ecuador. The bold red line shows the monthly mean temperature from 2000–2012 in comparison with monthly mean temperature for 2012 (red dashed line). The dark blue shading shows the monthly mean rainfall from 2000–2012 in comparison with monthly mean rainfall for 2012 (light blue shading). The red bars show monthly totals of confirmed dengue cases from 2012.

Study Area—Dengue fever is hyper-endemic in Guayaquil, Guayas Province (Figure 2). There is a pronounced seasonal peak in dengue transmission from February to May, which follows the onset of the rainy season (Figure 3). Guayaquil is a tropical coastal port city (pop. 2,350,915) [19], and the largest city in Ecuador. It is located on land that was previously mangrove forest, and is bounded by the Guayas River and Estero Salado, which are part of the Guayas Estuary, the largest estuary in the Latin American South Pacific [22]. Unplanned settlements have developed around the estuary, and therefore were not provided with municipal services. These populations are particularly vulnerable to environmental pollution and climate impacts during rainy and extreme events, such as El Niño. Therefore, these populations are also likely at greater risk of the persistence of vector breeding habitat, facilitating and sustaining dengue outbreaks.

Guayaquil is one of the centers of economic activity of the country. In 2010 the young population (<15 years of age) was 29% of the total population, and 65.4% were classified as adults (15 to 64). People move to Guayaquil from throughout Ecuador, seeking improved socio-economic conditions. According to the 2010 census, most of the population of Guayaquil is self-identified as mestizo (71%), white (11.4%), Afro-Ecuadorian (10.9%), Montubio or people from rural land (5%), indigenous (1.4%), and the rest from a variety of ethnic identities [19].

Data Sources—For the city of Guayaquil, we analyzed MoH dengue case reports for 2012 and social-ecological data from the national census, which was last conducted in 2010. These data were provided by INAMHI via a research collaboration with the MoH that was supported by the National Secretary of Higher Education, Science, Technology and Innovation (SENESCYT) of the Ecuadorian government from 2011 to 2013 [23]. All data were de-identified and aggregated to the census block level. As such, no formal ethical review was required. Figures were created in ArcGIS version 10.3.1 (ESRI, Redlands, CA, USA) [24] using shapefiles from the GADM database of Global Administrative Areas, version 2.8, freely available at gadm.org [25]. Inland rivers and water bodies data are derived from the Digital Chart of the World (DCW) [26]. Census block outlines were digitized by authors from the National Institute of Meteorology and Hydrology (INAMHI) during the course of this project.

Epidemiological records—For the analyses presented here, we analyzed de-identified georeferenced dengue cases from Guayaquil in 2012 (*n* = 4248), aggregated and mapped to census polygons [27] (Supplemental Data File S1). Cases included clinically diagnosed and laboratory confirmed cases of dengue fever. In Ecuador, dengue is a mandatory notifiable disease. Cases were reported to a surveillance system operated by the MoH, and included 15.03% of total dengue cases in Ecuador in 2012 (*n* = 16,544) [27]. 

Social-ecological risk factors—We identified variables from the 2010 national census [19] that have been previously described as dengue risk factors, and used in similar epidemiological studies [17] (Table 1). Individual-level and household-level data were extracted from the census for the city of Guayaquil. We created a normalized housing condition index (0 to 1, where 0 is the best) by combining three housing variables regarding the condition of roofs, walls, and floors. Census variables were recoded, and we calculated census block level variables (e.g., the proportion of homes or proportion of the population per census block) (*n* = 484 census blocks). 

Climate Data—INAMHI provided rainfall and 2-m temperature station data at monthly scale for the period 1981–2012. The long-term means were computed for both variables, and monthly values for the year 2012 were compared with those climatological values (Figure 3). We performed an additional analysis to better understand the behavior of these two variables during 2012, using sea-surface temperature fields from both the Pacific and the Atlantic Oceans (ERSST version 4 [28]), and vertically integrated moisture fluxes computed using the NCEP-NCAR Reanalysis Project version 2 [29]; these datasets are publicly available in the International Research Institute for Climate and Society (IRI) Data Library.

Statistical Analyses—To understand the spatial distribution of dengue transmission in the city of Guayaquil during the 2012 epidemic, we used Moran’s I with inverse distance weighting. We tested the hypothesis that dengue incidence was randomly versus non-randomly distributed across the census blocks. Hot and cold spots of dengue incidence were identified using Local Indicators of Spatial Association (LISA) methods, specifically Anselin’s Local Moran’s I with inverse distance weighting [30]. Analyses were conducted in ArcGIS (ver. 10.3.1, ESRI, Redlands, CA, USA) [24].

We analyzed social-ecological variables from the national census that we hypothesized were associated with dengue presence and burden (count of cases at the census block level) (Table 1). Two model searches were performed in R, using ‘glmulti’ for multimodel selection [31]. The first search was to determine which census factors were influencing the presence or absence of dengue in Guayaquil, specifying a logistic modeling distribution in a Generalized Linear Model (GLM) framework (GLM, family = binomial, link = logit). The second model search examined which census factors were influencing outbreak severity, defined as burden—dengue case counts per census block, offset by local population, as the dependent variable (GLM, family = negative binomial). Model searches were run until convergence using glmulti’s genetic algorithm (GA) [31]. Models were ranked based on Akaike’s Information Criterion corrected (AICc) for small sample size. The top ranked model for each search was compared to its respective global model, that contained all variables [32]. We calculated parameter estimates and 95% confidence intervals (CI) for the parameters in the top models from each search. We estimated variance inflation factors (VIF) to evaluate multi-collinearity and dispersion in the models. 

Similar methods have been used in prior studies to describe the distribution of dengue risk across the landscape [33], including in Ecuador [6].

## 3. Results

### 3.1. Spatial Analyses

**Figure 4:**
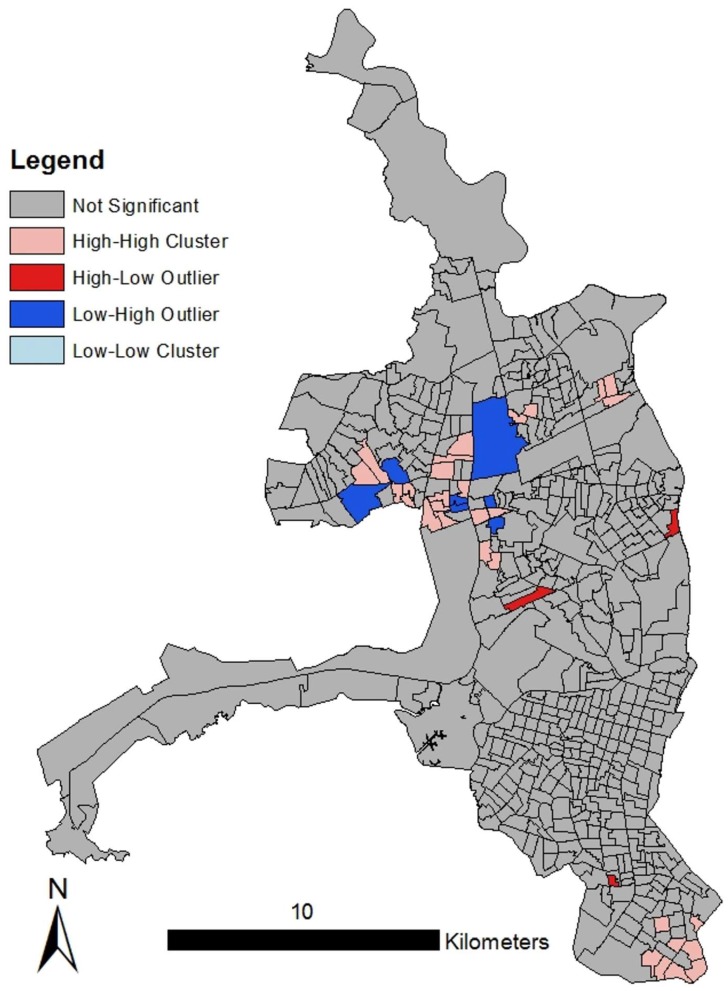
Anselin’s Local Moran’s I analysis for the 2012 Guayaquil outbreak. Cases of dengue were significantly clustered in the North Central and Southern areas of the city.

Dengue incidence in census zones ranged from 0 cases (*n* = 88 zones) to 160 cases per 10,000 population (*n* = 1 zone) (Figure 4). We found that dengue incidence was non-randomly distributed (e.g., clustered; Moran’s I = 0.066, *p* < 0.05). We identified dengue hotspots in the North Central and Southern areas of the city (*n* = 30 high-high census zones; *p* < 0.05, Figure 4). These clusters comprised clusters of high neighboring census zones (*n* = 30), with both high and low outlier zones, (high-low and low-high), indicating hotspots and heterogeneity. 

### 3.2. Social-Ecological Risk Factors

The most important factors associated with the presence of dengue cases were poor housing conditions (e.g., poor structural condition of the floor, roof, and walls) and the proportion of households that received remittances. Other significant risk factors included greater access to municipal services (sewerage, piped water, garbage collection), fewer households that drink tap water, and lower proportion of Afro-Ecuadorians in the local population (Table 2). Ten additional models were found within two AICc units of the top model (Table S1).

Poor housing condition was also the most important risk factor associated with the severity of localized dengue outbreaks in Guayaquil, as determined by dengue burden. Other risk factors included lower proportion of heads of household with postsecondary and primary education, lower proportion of Afro-Ecuadorians in the population, lower proportion of household members less than 15 years old, older age of the heads of household, greater access to municipal garbage collection, a greater proportion of housing structures with more than one household, and a greater proportion of families receiving remittances (Table 3). Twenty-nine additional models were found within two AICc units of the top model (Table S2).

Results from the VIF analysis showed that 17 of the 23 tested variables had VIF scores under 10, indicating a fair degree of collinearity among certain predictors within census categories (e.g., measures of education and household age structure showed some correlation). Collinear variables were included in the multimodel searches as the main concern with inflated VIF scores is large error terms, not the coefficient estimates. Collinear variable suites were shown to be significant in many top models even with conservative model search criteria in place. 

### 3.3. Climate Analysis

The 2012 outbreak occurred toward the end of a weak La Niña event (2011/2012), with a peak of reported dengue cases around March, just after the precipitation peak of February brought anomalously high rainfall (approximately twice as much as the typical values for Guayaquil), and concurrent with an increase in temperatures from below-normal to normal seasonal values (Figure 3). Although identified as a weak La Niña due to the behavior of the sea-surface temperature anomalies in the Equatorial Pacific (see Figure S1, “climate” a, c, d), and contrary to the expected situation of a typical La Niña event, anomalously high vertically-integrated moisture fluxes continuously arrived to coastal Ecuador from the Pacific during January and March (see Figure S1 “climate” b, d, e), providing suitable conditions for the above-normal rainfall amounts observed during the season in Guayaquil.

## 4. Discussion

Since the 1980s, the burden of febrile illnesses transmitted by *Ae. aegypti* and *Ae. albopictus* (i.e., dengue fever, chikungunya, Zika fever) has increased despite significant investment in vector control programs [2,10,34]. Targeted interventions and new surveillance strategies are urgently needed to halt the spread of these diseases. Our findings indicate the importance of differentiating between disease burden and presence when developing risk maps. This study also provides an important local-level characterization of transmission dynamics, which are complicated by the geographic and temporal variation in the intrinsic and extrinsic factors that drive disease transmission [12,13,14,15]. 

Spatial characteristics—During the 2012 outbreak, we identified hotspots of dengue fever transmission in the North Central and Southern areas of the city of Guayaquil, where land use is a mix of densely populated urban neighborhoods, industrial lots, and parks. Although they have access to basic municipal services, findings from nearby Machala, El Oro, another port city on the Southern Ecuadorian coast, indicated that some communities in the urban periphery in coastal Ecuador have weak social organization and limited interaction with local authorities [5]. During the period of this study, vector control in these areas consisted of larvicidal products distributed by public health workers, with the expectation that these products were applied by individual households. Although there has been no formal evaluation of public mosquito abatement, health workers have indicated that homeowners did not apply the larvicides. It should be noted that these census data do not capture the quality of the access to services, for example, the frequency of disruptions in the water supply or the frequency of garbage collection, which have a direct effect on mosquito larval habitat. Previous studies have also found significant clustering of dengue transmission in urban landscapes [12,35,36,37]. In Guayaquil, previous work also identified clustered dengue transmission. Hot and cold spots moved from year to year over a 5-year period, pointing to the importance of continued spatial surveillance, and tracking potential risk factor shifts [6,7]. Fine-scale clustering of dengue transmission in space and time has also been seen in Thailand [38,39,40,41], and in Peru, where urban spatial transmission dynamics have been linked to human movement patterns within the urban environment [42,43]. Given the reported dispersal range of the *Aedes aegypti* vector of approximately 250 m [6,7], we suggest that a combination of vector flight range, and intra-urban human movement, may lead to localized hotspot patterns, while enabling broad scale spread of dengue across Guayaquil. 

**Figure 5:**
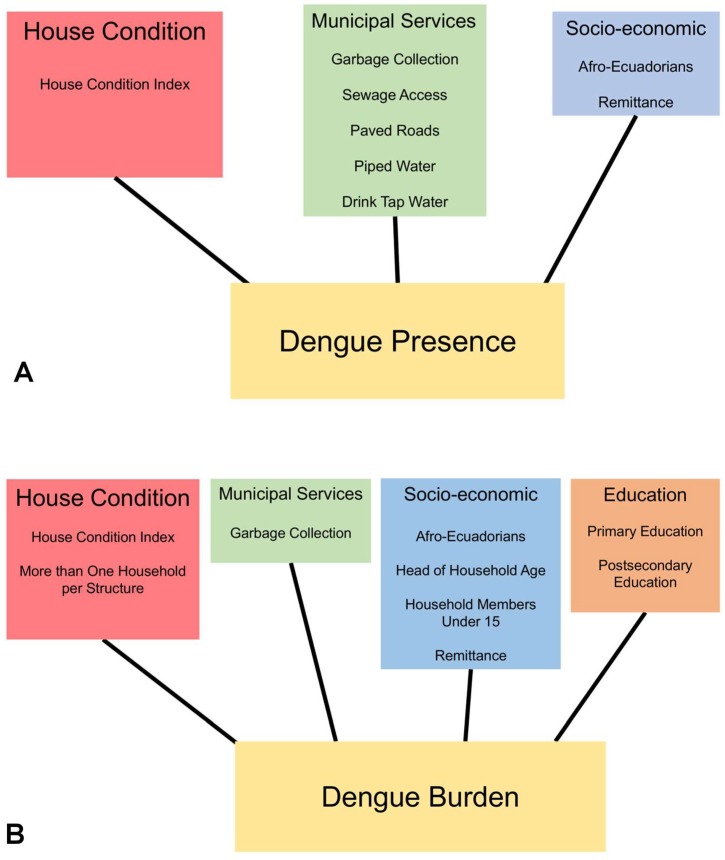
Conceptual diagrams highlighting the census variable suites that significantly affected dengue presence (**A**) and dengue burden (**B**) in Guayaquil, Ecuador during the 2012 outbreak.

Social-ecological risk factors—Poor housing condition was the variable most strongly associated with dengue transmission in Guayaquil, influencing both the presence/absence of dengue cases and the localized burden of the outbreak. Dengue was more likely to be present in a census block when housing structures (i.e., roofs, walls, and floors) were in poor condition, access to paved roads was limited, and the proportion of houses receiving remittances was high. The risk factors for higher dengue burden were poor housing condition, proportion of houses receiving remittances, and the number of dwellings housing more than one family. These results suggest that accessibility of households to mosquitoes via structural deficiencies, as well as the overall socioeconomic status of neighborhoods, played a role in the 2012 outbreak (Figure 5). Although the role of poverty in dengue transmission is not clearly defined, the relationship between poor housing structure and arbovirus transmission has been well documented [44,45,46,47]. Following the economic crisis in the late 1990s, many Ecuadorians immigrated to the U.S., Spain, and other countries in Europe for work, resulting in fragmented households and communities, and increasing reliance on remittances. The role of immigration in urban dengue control and prevention should be explored further [48,49,50].

**Figure 6:**
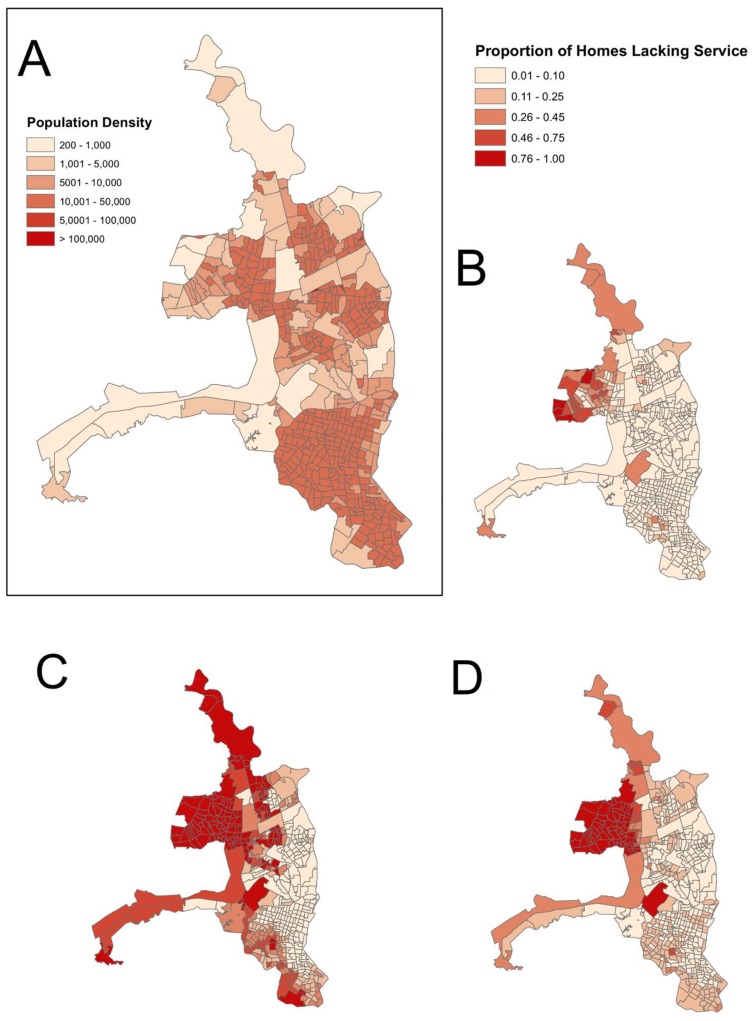
Population density (people per km^2^) of census zones in Guayaquil (**A**) shown against the proportion of homes lacking municipal garbage collection (**B**); lacking municipal sewage (**C**); and lacking piped water (**D**). Although dengue cases were reported in both densely and sparsely populated census zones, dengue hot spots were more associated with higher density zones (Figure 1), and the proportion of homes that lack basic municipal services tends to be higher in zones with lower population density. This may account for the counterintuitive model estimates associated with lack of these services (Table 2 and Table 3).

When modeling the presence of dengue, all top models included access to core municipal services such as garbage collection, sewage, access to piped water, and number of houses drinking tap water as positive predictors of dengue cases (Table 2, Table S1). Municipal garbage collection was also positively correlated with dengue burden in all top models (Table 3, Table S2). Previous studies in smaller communities have observed positive correlations between lack of services and dengue transmission, as poor sanitation and water storing habits in urban areas are well-documented for providing habitat for larval Aedes mosquitoes [16,17]. Although municipal services are known to reduce the amount of larval mosquito habitat, there is some evidence to suggest that heavily urbanized areas, like Guayaquil, provide ample habitat regardless of service availability [51]. Municipal services in Guayaquil are spatially heterogeneous, but in general services are more widely available in densely populated areas of the city (Figure 6). However, access to service does not necessarily serve as an indicator for quality or frequency of services. Several studies have identified the interaction between local *Aedes* production and human population density as a key factor in triggering dengue outbreak events [12,51,52,53]. The observed counterintuitive findings may indicate that although access to services should reduce the amount of available habitat for larval mosquitoes, human population density and quality of services may be more important. Intermittent or interrupted service may in fact exacerbate local conditions for mosquito breeding habitat, by increasing standing refuse piles, or prolonging duration of standing refuse, and in the case of water, increase problematic storage habits. Data on the quality of access to piped water was not available in the census.

Several demographic characteristics were negatively correlated with dengue incidence, i.e., age structure of households and access to primary and secondary education. Education, specifically knowledge about dengue, has been shown to influence the prevention practices of households and elimination of mosquito breeding sites [54]. Previous work in Machala, Ecuador, also revealed that household-level risk factors and perceptions of dengue risks vary with social and economic structures between communities [5]. The proportion of Afro-Ecuadorians per census zone was associated with both lower dengue presence and burden, indicating the possibility of cultural and racial differences influencing localized transmission, disproportionate case reporting, or differences in the clinical presentation of dengue infections in the Afro-Ecuadorian population. 

Our findings on the risk factors for dengue transmission in Guayaquil support findings from prior field studies of *Ae. aegypti* and household risk factors in the neighboring city of Machala, Ecuador. This study found that during the rainy season, *Ae. aegypti* pupae were more likely to be found in homes with poor house and patio conditions, and that during the dry season, *Ae. aegypti* were more likely to be found in homes with interruptions in the piped water supply [16]. Water storage in containers other than cisterns or covered elevated water tanks was a risk factor year-round. While the Machala study was in a smaller city, a different year, and different climate conditions, together these studies indicate the potential to target high-risk households for vector control and dengue case management, using rapid household surveys that have been locally tested and adapted. 

The model selection framework used in this study is an effective strategy for exploratory studies to capture a large number of complex social-ecological processes. In contrast to traditional frequentist statistical approaches, a model selection approach enabled us to test multiple hypotheses simultaneously and identify potentially important variables for inclusion, not limited to significant variables determined by arbitrary *p*-values, or excluded due to collinearity before testing. Information theoretic or likelihood modeling approaches allow the modeler, who has a priori knowledge of the system, to make explicit informed decisions about which variables to include when testing the model, and explore multiple compatible hypotheses rather than being limited to testing and excluding individual competing hypotheses. Additionally, the model search algorithms in the R package ‘glmulti’, facilitating exploration of all subsets of all possible models, is a more robust model selection procedure than stepwise regression techniques, which can lead to biased estimates [31,32]. 

Guayaquil is a large, heterogeneous urban area, and there may be reporting bias of dengue cases especially in less populated areas with reduced access to medical care. However, reporting bias may not be as profound in Guayaquil as in other less-developed coastal cities in Ecuador. While dengue incidence was highest in densely populated census zones, cases were consistently reported throughout most of the city (Figure 1). These findings support prior studies that showed spatio-temporal heterogeneity in dengue transmission across Guayaquil [6,7]. 

*Climate conditions*—Rainfall excess in 2012 produced moisture-saturated soils, formation of ponds of different sizes, water accumulation in a variety of containers, and other suitable conditions for vector proliferation. The transition to higher temperatures between February (rainfall maximum) and March is hypothesized to have contributed to the outbreak, as was the case for an analysis of similar conditions for a dengue outbreak in Machala in 2010 [15]. 

## 5. Conclusions

The results of this study indicate that certain social-ecological factors were associated with the presence/absence and burden of dengue during the 2012 epidemic in the city of Guayaquil, Ecuador. We also demonstrated that the number and magnitude of variables influencing the presence versus the burden of dengue were fundamentally different, although some similarities were noted. The most important risk factors for both presence and increased burden of dengue during the outbreak included poor housing condition and the proportion of households receiving remittances. Dengue transmission was spatially clustered at the sub-city level, highlighting areas for investigation into underlying causes of risk in these areas. Spatial clustering of dengue cases within Guayaquil and identification of social-ecological drivers for disease presence and burden indicate the potential to develop operational rapid survey tools to target high-risk households, and the importance of spatially explicit analyses to inform surveillance and intervention efforts. These findings contribute to a long-term collaboration with INAMHI and the MoH to strengthen arbovirus surveillance and response systems in the region [8]. 

## Figures and Tables

**Table 1:** Socio-ecological parameters tested in logistic regression and negative binomial model searches to respectively predict presence of dengue and severity of the outbreak.

Parameter	Mean	SD
Housing conditions		
House condition index (HCI), 0 to 1, where 1 is poor condition	0.27	0.12
More than four people per bedroom	16.78%	0.08
People per household	3.88	0.34
Municipal garbage collection	93.06%	0.15
People in household drink tap water	76.85%	0.09
Piped water inside the home	77.03%	0.31
Municipal sewage	62.52%	0.39
Access to paved roads	80.06%	0.25
More than one household per structure	1.90%	0.01
Unoccupied households	16.08%	0.56
Rental homes	1.55%	0.17
**Demographics**		
Receive remittances	8.85%	0.04
People emigrate for work	1.88%	0.01
Mean age of the head of the household (years)	45.69	4.54
Mean household age (years)	29.36	4.29
Proportion of household under 15 years of age	28.34%	0.06
Proportion of household under 5 years of age	9.31%	0.03
Head of the household has primary education or less	30.94%	0.15
Head of household has secondary education	31.73%	0.07
Head of household has post-secondary education	25.77%	0.21
Afro-Ecuadorian	10.13%	0.07
Head of the household is unemployed	26.84%	0.06
Head of the household is a woman	33.29%	0.04

**Table 2:** Top logistic regression model used in determining which social-ecological factors are important to dengue presence.

Model	Estimate	95% CI	SE	AICc	*p*-Value
Intercept	3.84	0.54–7.25	1.71	369.85	0.03
House condition	24.55	17.62–32.11	3.69		<0.001
Proportion of Afro-Ecuadorians	−9.69	−15.72–−3.76	3.04		0.001
Municipal garbage collection	4.70	2.27–7.37	1.29		<0.001
Piped water	3.50	1.38–5.72	1.10		0.002
Municipal sewage	2.04	0.44–3.62	0.81		0.012
Access by paved roads	−3.36	−6.36–−0.54	1.48		0.023
Drink tap water	−10.74	−16.53–−5.28	2.86		<0.001
Remittance	23.20	10.83–36.15	6.44		<0.001

**Table 3:** Top negative binomial model used in determining which social-ecological factors are important to dengue burden.

Model	Estimate	95% CI	SE	AICc	*p*-Value
Intercept	1.04	−4.09–6.25	2.54	2920.67	0.682
House condition	10.95	6.77–15.13	2.09		<0.001
Postsecondary education	−2.53	−4.72–−0.34	1.07		0.018
Primary education	−5.11	−8.52–−1.71	1.62		0.002
Proportion of Afro-Ecuadorians	−4.23	−6.43–−1.94	1.25		<0.001
Proportion of household members under 15	−9.02	−15.58–−2.50	3.46		0.009
Head of household age	−0.12	−0.19–−0.05	0.03		<0.001
Municipal garbage collection	2.82	1.77–3.87	0.61		<0.001
More than 1 household per structure	7.57	−4.66–20.03	6.26		0.227
Remittance	4.76	−1.27–10.87	3.07		0.121

## Supplementary Materials

The following are available online at http://www.mdpi.com/1660-4601/15/4/827/s1, Figure S1: Sea-surface temperature anomalies (contours (°C)) and surface-level winds (vectors (m/s)) (panels a, c, e); vertically-integrated moisture flux anomalies (g/kg m/s) over Ecuador (panels b,d,f), during January, February and March 2012 (top, middle and bottom rows, respectively). Red dot indicates location of Guayaquil, Table S1: All logistic regression models for presence or absence of dengue cases ≤2 AICc units, Table S2: All negative binomial regression models for burden of dengue cases ≤2 AICc units, Data File S1: 2012 dengue case data aggregated to census block level, Guayaquil, Ecuador, provided as a polygon shapefile created in ArcGIS version 10.3.1 [29]. Census block outlines were digitized by INAMHI (National Institute of Meteorology and Hydrology) authors during the course of this project, as reported in the methods.
